# Effect of Different Post-Curing Methods on the Degree of Conversion of 3D-Printed Resin for Models in Dentistry

**DOI:** 10.3390/polym16040549

**Published:** 2024-02-18

**Authors:** Scott Kirby, Igor Pesun, Anthony Nowakowski, Rodrigo França

**Affiliations:** 1 Graduate Prosthodontics Program, Department of Restorative Dentistry, Dr. Gerald Niznick College of Dentistry, University of Manitoba, Winnipeg, MB R3E 0W2, Canadaigor.pesun@umanitoba.ca (I.P.); 2 Department of Restorative Dentistry, Dr. Gerald Niznick College of Dentistry, University of Manitoba, Winnipeg, MB R3E 0W2, Canada; anthony.nowakowski@umanitoba.ca; 3 Dental Biomaterials Research Laboratory, Department of Restorative Dentistry, Dr. Gerald Niznick College of Dentistry, University of Manitoba, Winnipeg, MB R3E 0W2, Canada

**Keywords:** additive manufacturing, post-curing 3D printing, degree of conversion

## Abstract

The aim was to investigate the effects of different post-curing units on the chemical properties (degree of conversion) of 3D-printed resins for producing models in dentistry. The goal is to determine whether less-expensive post-curing units can be a viable alternative to the manufacturer’s recommended units. Forty-five samples were fabricated with an LCD printer (Phrozen Sonic Mini, Phrozen 3D, Hsinchu City, Taiwan) using MSLA Dental Modeling Resin (Apply Lab Work, Torrance, CA, USA). These samples were divided randomly into four different groups for post-curing using four distinct curing units: Phrozen Cure V2 (Phrozen 3D, Hsinchu City, Taiwan), a commercial acrylic nail UV LED curing unit (SUNUV, Shenzhen, China), a homemade curing unit created from a readily available UV LED light produced (Shenzhen, China), and the Triad^®^ 2000™ tungsten halogen light source (Dentsply Sirona, York, PA, USA). The degree of conversion was measured with FTIR spectroscopy using a Nicolet 6700 FTIR Spectrometer (Thermo Fisher Scientific, Waltham, MA, USA). Phrozen Cure V2 had the highest overall mean degree of conversion (69.6% with a 45 min curing time). The Triad^®^ 2000 VLC Curing Unit had the lowest mean degree of conversion value at the 15 min interval (66.2%) and the lowest mean degree of conversion at the 45 min interval with the homemade curing unit (68.2%). The type of light-curing unit did not yield statistically significant differences in the degree of conversion values. There was a statistically significant difference in the degree of conversion values between the 15 min and 45 min curing intervals. When comparing individual light-curing units, there was a statistically significant difference in the degree of conversion for the post-curing units between the 15 min and 45 min curing time (*p* = 0.029).

## 1. Introduction

Digital dentistry has become increasingly popular in recent years among private practitioners, researchers, and educational institutions. At the heart of digital dentistry, a significant amount of attention has been focused on computer-aided design and computer-aided manufacturing, commonly referred to as CAD/CAM. The computer-aided-design (CAD) side of the workflow in dentistry initially involves a method of digitizing data, such as a desktop or intraoral scanner to capture a digital impression, and software to manipulate that data and design a myriad of different restorations or appliances [1]. The computer-aided-manufacturing (CAM) portion of the workflow can be broken down into two basic processes including subtractive manufacturing and additive manufacturing [2].

Additive manufacturing, commonly known as 3D printing, has become an extremely popular topic of interest in recent years, particularly within the dental community. There are currently seven main categories of additive-manufacturing techniques which include the following: vat photopolymerization, material jetting, material extrusion or fused deposition modeling, binder jetting, powder bed fusion, sheet lamination and direct energy deposition [3]. Within dentistry, the most commonly used methods of additive manufacturing are vat photopolymerization and material jetting [2]. Many of the same materials used in a subtractive manufacturing process can also be used in an additive manufacturing fashion but the nature of 3D printing lends itself to be less wasteful due to the fact that the only material used is the material needed to produce the object [4].

One of the main differences between a subtractive and an additive approach is the rigorous post-processing step that is necessary for 3D-printed objects which is generally not required in the subtractive method [5,6,7,8,9,10,11,12,13,14,15]. Post-processing methods are crucial in 3D printing with polymers to enhance the overall quality and performance of printed objects. While 3D printing offers numerous advantages, such as complex geometries and rapid prototyping, the printed parts often require additional treatment to achieve desired properties. The most common post-processing methods are to improve the surface finishing (sanding, polishing, or chemical treatments), to improve dimensional accuracy (heat treatment and annealing), and to improve strength and durability (post-curing). The post-curing process has the goal of increasing the degree of conversion (DC), which refers to the extent to which a polymer resin has undergone a chemical reaction and transformed into a solid polymer during the 3D-printing process [16]. The higher the DC the lower the number of residual monomers (that could act as plasticizers) and the higher the mechanical properties [7,8,9,10,11,12,13,14,15,16,17,18].

In general, each type of 3D-printing technology and individual printer may have its own manufacturer’s recommendations for post-processing and post-curing [6]. The main features of a post-curing oven may vary depending on the specific model or manufacturer, but some essential features are as follows: (a) UV exposure: Post-curing ovens often utilize Ultra Violet (UV) light to initiate and accelerate the curing process of photopolymer resins used in 3D printing. It is commonly used in 3D-printing techniques such as SLA (Stereolithography) and DLP (Digital Light Processing. This exposure may come from one or many LED units to enhance curing and improve the mechanical properties of the printed parts. (b) Heating capability: Some post-curing ovens also incorporate heating elements to provide controlled temperature conditions during the curing process. Typically, post-curing ovens equipped with high-power bulbs offer additional heating capabilities. (c) Adjustable settings: Many post-curing ovens may include a turntable as a standard feature, allowing users to adjust the curing time, temperature, and UV intensity according to their specific requirements. This flexibility enables the customization and optimization of the post-curing process for different resin materials and desired outcomes [5,6].

There appears to be a lack of research examining the impact of correct or incorrect post-processing methods on the properties of 3D-printed materials, especially those produced using vat photopolymerization. Given the high expense of dental-specific post-processing units, there is a trend among dentists to approach the post-processing stage with a “DIY” mentality, which includes using less-expensive alternative curing units. This off-label use of alternative curing units may not meet the criteria set by the manufacturer for the specific materials used. Therefore, the degree of conversion is an ideal parameter to measure the effectiveness of different post-curing units. This study aims to investigate the effects of different post-curing units on the degree of polymerization of 3D printed resins to determine whether less-expensive post-curing units can be a viable alternative to the manufacturer’s recommended units. Our first null hypothesis states that there are no differences in the DC between the manufacturer’s recommended curing unit for the 3D-printed resin and the alternative curing units investigated. The purpose of testing this null hypothesis is to determine whether the choice of curing unit has a statistically significant effect on the degree of conversion of the 3D-printed resin. If the null hypothesis is rejected, it would indicate that there is a significant difference in the degree of conversion between the different curing units. The second null hypothesis posits that there are no disparities in the DC between 15 min and 45 min curing-time intervals. This specific time frame was selected to ensure that it does not surpass the final setting time of traditional gypsum casts.

## 2. Materials and Methods

A standardized geometric shape was designed using free CAD software, Meshmixer (Autodesk Research, Mill Valley, CA, USA), with dimensions of 10 × 4 × 2.5 mm. The sample design was exported in the standard tessellation language (STL) format and prepared for printing using the free slicing software Chitubox (CBD-Tech, Shenzhen, Guangdong, China). A total of 45 samples were fabricated with an LCD printer (Phrozen Sonic Mini, Phrozen 3D, Hsinchu City, Taiwan) using MSLA Dental Modeling Resin (Apply Lab Work, Torrance, CA, USA). The samples were printed directly on the build plate in groups of 5 in a 0° orientation to ensure even exposure to the LCD screen and limit discrepancies when testing the samples. The printing parameters used included a layer thickness of 100 µm, six bottom burn-in layers with 30 s exposure time for the burn-in layer, and a 6 s normal layer exposure time, as per the manufacturer’s recommendations (Apply Lab Work, Torrance, CA, USA). The samples were then subjected to a two-step alcohol wash with 85% ethyl alcohol for four minutes in a preliminary rotary alcohol wash unit (Anycubic Wash and Cure, Anycubic 3D, Shenzhen, China) to remove the bulk of the uncured resin and for one minute in the second final stationary wash of 85% ethyl alcohol to remove any remaining resin.

Post-curing of the samples was performed using four distinct curing units: 1. the Phrozen Cure V2 (PC) that is recommended by the 3D printer company; 2. the SUNUV (NC) a commercial acrylic nail UV LED; 3. the Triad^®^ 2000™ (TC) tungsten halogen light source; 4. A homemade curing oven (HC) fabricated from a readily available UV LED light source. The main technical features of the light-curing units are displayed in Table 1. A sample size of *n* = 45 was used based on power calculations for dental-material testing, which yielded reasonable confidence limits with a small bias [8]. Post-curing was performed at two separate time intervals: 15 min and 45 min. Five samples were assigned to each of the four curing unit groups at each curing-time interval. In addition, five samples collected directly from the printer and not subjected to any post-curing methods were also included in the study as a baseline reference.

**Figure 1:**
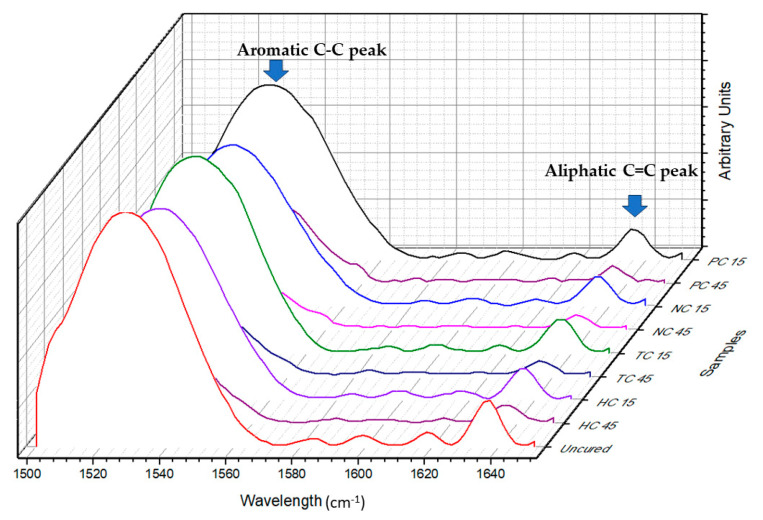
FTIR spectra from 1500 cm^−1^ to 1650 cm^−1^ for all tested groups compared with uncured baseline.

Fourier-transform infrared spectroscopy (FTIR) analysis was performed to analyze the difference in the functional groups of the resin using a Nicolet 6700 FTIR Spectrometer (Thermo Fisher Scientific, Waltham, MA, USA). Infrared spectra in the range of 400–4000 cm^−1^ were generated from 120 scans for each sample, beginning with unpolymerized liquid resin as a baseline, followed by samples from each test group. These spectra were analyzed in the absorbance mode, and the values corresponding to the aliphatic C=C peak (1637 cm^−1^) and aromatic C-C peak (1525 cm^−1^) before post-processing and after post-processing were used to determine the degree of conversion [9]. An example spectrum is portrayed in Figure 1. OMNIC^TM^ Spectra Software (Thermo Fisher Scientific, Waltham, MA, USA) was used to add baseline correction for each spectrum. The degree of conversion was calculated using the formula shown in Equation (1). The data were analyzed statistically using two-way analysis of variance (ANOVA) followed by Tukey’s post hoc test. A significance level of α = 0.05 was used for all statistical analyses.

(1) $$\mathrm{DC}\%=1-\left[\frac{C_{\mathrm{aliphatic}}/C_{\mathrm{aromatic}}}{U_{\mathrm{aliphatic}}/U_{\mathrm{aromatic}}}\right]\;\times \;100$$

Equation (1). Degree of conversion equation. C_aliphatic_ represents the peak intensity of the Aliphatic carbons in the cured samples. C_aromatic_ represents the peak intensity of the Aromatic carbons in the cured samples. U_aliphatic_ represents the peak intensity of the Aliphatic carbons in the uncured samples. U_aromatic_ represents the peak intensity of the Aromatic carbons in the uncured samples.

## 3. Results

**Figure 2:**
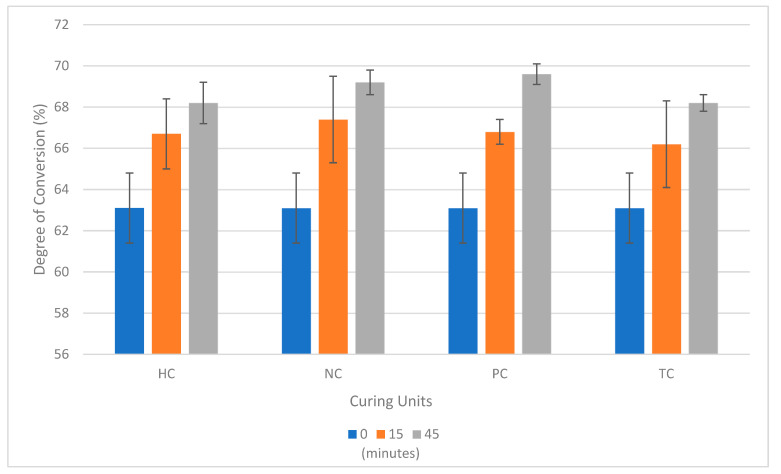
Mean and standard deviation for the degree of conversion (%) for HC = homemade curing unit, NC = SUNUV nail curing unit, PC = Phrozen Cure V2, TC = Triad^®^ 2000 VLC Curing Unit.

The mean values for the degree of conversion and their standard deviations are graphically represented in Figure 2. The values for 0 min were calculated by measuring the degree of conversion on the samples straight from the printer that did not undergo any post-curing for use as a baseline reference. Phrozen Cure V2 had the highest overall mean degree of conversion (69.6%) with a 45 min curing time. The Triad^®^ 2000 VLC Curing Unit had the lowest mean degree of conversion value at the 15 min interval (66.2%) and the lowest mean degree of conversion at the 45 min interval with the homemade curing unit (68.2%).

Based on the two-way ANOVA test, the type of light-curing unit did not yield statistically significant differences in the degree of conversion (*p* = 0.171); however, there was a statistically significant difference in the mean degree of conversion between the 15 min and 45 min curing-time intervals (*p* < 0.001). Tukey’s post hoc test indicated that when comparing individual light-curing units, there was a statistically significant difference in the degree of conversion for the Phrozen Cure V2 between the 15 min and 45 min curing time (*p* = 0.00023).

## 4. Discussion

This study evaluated the effects of alternative light-curing units on the degree of conversion of a 3D-printed dental model resin at two common curing-time intervals. The results of this study showed there was no statistically significant difference in the degree of conversion values between light-curing units, which led to the acceptance of the first null hypothesis. (*p* = 0.171) However, it also showed a statistically significant difference in the degree of conversion values between the 15 and 45 min curing-time intervals and, therefore, rejected the second null hypothesis (*p* < 0.001).

It is worth noting that the models generated using the 3D printer initially have a low DC (63.1%). It can be explained through the kinetics of the polymerization reaction. Kinetic polymerization is a process in which monomers undergo a series of chemical reactions to form polymer chains. At the beginning of the reaction, monomers are in a liquid-like state, capable of freely moving and reacting with other monomers. This high mobility allows for efficient diffusion of reactive species, promoting polymerization and contributing to the exponential increase of the DC until the gel point. The gel point refers to the point in the polymerization reaction where the polymer chains become entangled and form a three-dimensional network known as a gel. At this point, the reaction transitions from a liquid-like state to a solid-like state. After the gel point, the polymer network begins to form, resulting in a transition from a liquid-like state to a gel-like state. At this stage, the monomer mobility decreases significantly as the polymer chains start to crosslink and form a three-dimensional network [9]. The gel point marks the point at which the polymer network becomes self-supporting and exhibits a solid-like behavior. The decrease in monomer mobility after the gel point can impact the DC. The mobility of unreacted monomers is limited after the gel phase, making it more difficult for them to diffuse and react with other monomers. In stereolithography 3D printing, resins are cured in layers that are typically between 25–100 μm thick, using a high-intensity light source that can cure each layer in just a few seconds. However, this rapid curing process can result in a short pre-gel phase with reduced mobility, which can lead to incomplete conversion and a lower overall degree of conversion.

The post-curing process was designed to increase the DC of the 3D-printed objects and consequently enhanced the material properties and dimensional accuracy. The literature suggests that post-curing can significantly influence the mechanical properties of the final product by optimizing the light intensity and duration of exposure during the process. For example, one study indicated that the light intensity of the post-curing device plays a crucial role in determining the final mechanical properties of 3D-printed dental products, suggesting that more efficient post-curing can be achieved by fine-tuning these parameters [19]. The review of 3D-printing technologies in dentistry also emphasizes the importance of post-curing in achieving high material utilization and producing complex geometries with high accuracy [19,20,21]. However, our results showed an increase of ~ 10% of DC after 45 min of the post-curing process for all kinds of ovens. This timid result can be explained as due to the decreased monomer mobility that can lead to diffusion limitations within the polymer network. This can hinder the penetration of light or other curing agents in the resin, affecting the depth of cure. It is important to note that the specific impact of monomer mobility on the degree of conversion can vary depending on factors such as resin formulation, curing conditions, and the specific curing mechanism employed by the light-curing unit. These factors can interact with monomer mobility to influence the overall conversion efficiency and final degree of conversion achieved. The degree of conversion values found in this study fell within the normal range of 3D-printed resins available currently in the scientific literature; however, to the best of our knowledge, most of the studies available on the degree of conversion have investigated prosthodontic provisional 3D-printed resins, not dental model resin [10]. When compared to other photopolymerized resins used in dentistry, including dental adhesives and orthodontic bonding resin, even though the object size and shape were different, the degree of conversion values in this study were also comparable [11,12].

Another parameter that can affect the properties of 3D-printed resins is the layer thickness. A study that investigated layer thickness and degree of conversion found that for objects tested directly from the printer without post-curing a layer thickness of 25 μm had the highest values of degree of conversion, followed by 50 μm and 100 μm [13]. However, once these objects were post-cured, the highest values for degree of conversion were found in the samples printed with 100 μm and 50 μm layer thickness with 25 μm samples having the lowest degree of conversion [13]. This phenomenon may be explained by the “over-curing” that potentially can happen when the light source penetrates deeper than the layer thickness, which can, in turn, lead to inaccuracies as well as excess heat generation that has the potential to negatively impact polymerization at the single-layer level [14,15]. The layer thickness used in the printing of the samples used in this study was 100 μm, which appears to be the optimal layer thickness from the degree of conversion standpoint but also from a practicality standpoint as decreased layer thickness leads to longer print times and sometimes unnecessary accuracy depending on the dental application.

The duration of exposure during the post-curing process plays a crucial role in directly impacting the mechanical properties and overall performance of the final product. An extension of the post-curing time can lead to increased strength, rigidity, and overall durability of the resin parts. This is because prolonged exposure to UV light or heat allows for more thorough polymerization of the resin, providing additional energy to the residual monomers and resulting in a denser cross-linked network within the material [10,11,12,13,14,15,16,17,18,19,20,21,22] This study revealed a statistically significant variance in DC values between 15 min and 45 min curing intervals (*p* = 0.00023), supporting the idea that longer post-curing times result in higher degrees of conversion. However, even with a three-fold increase in time, there was only a minor, less than 5%, improvement in DC. Further investigations and advancements are required to achieve DC values approaching the desired level of 95%.

The main contribution of this study was to demonstrate that many of the “essential requirements” for a post-curing light-curing unit lack scientific evidence. The results indicated that there were minimal and statistically insignificant differences in the degree of conversion values among the four light-curing units tested. Table 1 highlighted the differences among the four ovens such as the kind of light bulb, the number of LEDs, the presence of a turning platform, and the power of the light source, and all these features did not significantly impact the DC. Interestingly, the study found that curing samples for 45 min with the Phrozen Cure V2, which is recommended by the 3D-printer manufacturer, resulted in the highest overall mean degree of conversion value (69.6%). On the other hand, the Triad^®^ 2000 VLC Curing Unit (using a tungsten halogen bulb) and the homemade curing unit yielded DC mean values at the 15 min interval (66.2%) and 45 min interval (68.2%), respectively. Furthermore, it was commonly believed that a turning platform within a post-curing oven is crucial for achieving an even and consistent cure for resin-based prints in the 3D-printing process. However, the samples from group NC, which used a SUNUV oven without a rotating plate, contradicted this assumption by showing the same level of DC (69.2% after 45 min).

In conclusion, based on the results of this study, it can be inferred that any of these curing units can yield a similar degree of conversion in common practice. These findings challenge the notion of certain “essential requirements” for post-curing light-curing units and emphasize the need for further scientific evidence in this area.

One of the limitations of this study is that it was performed during the global pandemic COVID-19 when supply chains of isopropyl alcohol were extremely compromised for 70% IPA (99%). The solvent used in this study was 85% ethyl alcohol, which is a common solvent for rinsing 3D-printed objects in countries where isopropyl alcohol is not readily available due to safety concerns regarding the flammable nature of the solvent. However, to the best of our knowledge, there are no scientific studies comparing ethyl alcohol as a solvent in the post-processing of 3D-printed resins which could have affected the results of this study with regard to the degree of conversion values.

Another limitation of this study is that it did not include one of the light-curing units that cures under an oxygen-deprived–nitrogen-rich atmosphere. None of the curing units investigated in this study controlled the environment under which they were cured. As previously mentioned, curing under a nitrogen-rich environment may result in higher number of conversion values and may have been beneficial to investigate these types of units and compare them to the alternative curing units investigated in this study [13].

Most 3D-printed resins available currently have special formulations and secret-patented compositions that make analysis difficult. In this study, the dental model resin used from ApplyLabWork did not disclose its exact chemical composition, which proved to be challenging when analyzing the specific functional peaks for FTIR spectroscopy. In the current study, the 1525 cm^−1^ wavenumber was used for the analysis of the aromatic C-C functional group, as opposed to other 3D-printed resins that use the 1608 cm^−1^ peak, which could be a source of discrepancy when comparing the literature.

The literature has already shown that the correlation between the degree of conversion and the mechanical properties of 3D-printed polymers is an important aspect of materials science, especially in applications where the mechanical integrity of the printed object is critical, such as in dental casts or medical devices. A higher degree of conversion typically indicates a more complete polymerization reaction, resulting in a denser cross-linked network within the material. This denser network can enhance the mechanical properties of the material, including its strength, Young’s modulus, and hardness [23,24,25].

It is important to acknowledge that the results presented in the manuscript may have limitations when applied to different applications. Factors such as printing layers and thickness, object dimensions, resin composition, curing waves, and curing powers can indeed influence the outcomes. Therefore, further rigorous inspection is necessary to determine the generalizability of the results.

In the future, it would be beneficial to investigate the effects of these alternative curing units with regard to the physical characteristics of this dental model resin, perhaps correlating the degree of conversion values with the flexural strength or hardness of the material. Another future consideration would be to investigate the effect of these alternative curing units on the degree of conversion values found within the internal aspects of the sample specimens, not just the superficial layers of 3D-printed objects. Also, further experiments with longer curing times are certainly warranted to determine the optimal curing time. Although the manufacturers’ recommendations are considered the gold standard for 3D-printing protocols, further studies are needed to investigate how different variables affect the outcomes of 3D printing in the dental field.

*Clinical implications:* The clinical implications of the findings from this study are significant for dental professionals. The study revealed that the choice of alternative light-curing units did not have a statistically significant effect on the DC of 3D-printed dental model resins. This suggests that dental professionals can have confidence in using different light-curing units (even a DIY device) without compromising the polymerization quality of the printed models. This finding provides flexibility in selecting light-curing units based on factors such as cost, availability, or personal preference. It can potentially reduce the cost barrier for dentists incorporating 3D-printed models into their practices.

However, the study also highlighted the importance of curing-time intervals. It demonstrated a statistically significant difference in DC values between the 15 and 45 min curing-time intervals. This implies that dental professionals should carefully consider the duration of the curing process when 3D printing dental models. Longer curing times may result in higher DC values, indicating more complete polymerization and potentially improved mechanical properties of the printed models. Therefore, it is crucial to adhere to recommended curing times to ensure optimal polymerization and the desired physical characteristics of the printed dental models.

Overall, these findings have clinical implications for dental professionals involved in the 3D printing of dental models. They provide insight into the choice of light-curing units and emphasize the importance of appropriate curing-time intervals to ensure optimal polymerization and mechanical properties of the printed models. By understanding these factors, dental professionals can enhance the accuracy, reliability, and longevity of 3D-printed dental models, ultimately improving patient outcomes and satisfaction.

## 5. Conclusions

The results from this study highlight that although there were slight differences between the investigated curing units, the overall degree of conversion values were comparable. Both the dental-specific light-curing units investigated, Phrozen Cure V2 and Triad^®^ 2000 VLC Curing Unit, were comparable in the context of the degree of conversion and level of post-curing efficacy to the off-label SUNUV Nail curing unit and even a homemade light-curing unit, easily fabricated from readily available components. In addition, curing-time intervals have a positive effect on the degree of conversion values and should be carefully determined depending on the specific resin and its application within the context of dentistry.

## Figures and Tables

**Table 1:** Technical specifications of the light-curing oven used in the experiment.

Post-Curing Oven		Specification	Manufacturer
PC	Phrozen Cure V2	UV-LED frequencies: 365 nm, 385 nm, 405 nm, 60 W. Plate: 360° rotation Voltage: 100–240 V 50–60 Hz	Phrozen 3D, Hsinchu City, Taiwan.
NC	SUNUV	39 separate UV-LEDs. Frequency: 365 nm and 405 nm and 48 W Plate: No rotation Voltage: 100–240 V 50–60 Hz	Sunuv, Shenzhen, China.
TC	Triad^®^ 2000™	One Tungsten halogen light in the range of 400 to 500 nanometers for visible light, 75 to 100 W. Plate: 360° rotation Voltage: 100–240 V 50–60 Hz	Dentsply Sirona, York, PA, USA.
HC	Homemade curing oven	20 UV LEDs ^a^ with 405 nm of wavelength inside a metallic cabinet ^b^ lined with reflective aluminum tape ^c^ and with rotating solar turntable ^d^.	^a^ Shenzhen Leader UV Technology, Shenzhen, China. ^b^ LIXHULT, IKEA, Smaland, Sweden. ^c^ Reflectix, Markleville, IN, USA. ^d^ Sovol 3D, Paris, France.

## Data Availability

The data presented in this study are available on request.

## References

[1] Beuer F., Schweiger J., Edelhoff D. (2008). Digital dentistry: An overview of recent developments for CAD/CAM generated restorations. Br. Dent. J..

[2] Sulaiman T.A. (2020). Materials in Digital Dentistry: A Review. J. Esthet. Restor. Dent..

[3] ASTM International (2013). F2792-12a Standard Terminology for Additive Manufacturing Technologies. Rapid Manuf. Assoc..

[4] Bae E.J., Jeong I.D., Kim W.C., Kim J.H. (2017). Comparative study of additive and subtractive manufacturing for dental restorations. J. Prosthet. Dent..

[5] Oberoi G., Nitsch S., Edelmayer M., Janjic K., Müller A.S., Agis H. (2018). 3D printing encompasses the facets of dentistry. Front. Bioeng. Biotechnol..

[6] Revilla-León M., Özcan M. (2019). Additive Manufacturing Technologies Used for Processing Polymers: Current Status and Potential Application in Prosthetic Dentistry. J. Prosthodont..

[7] Franz A., König F., Lucas T., Watts D.C., Schedle A. (2009). Cytotoxic effects of dental bonding substances as a function of degree of conversion. Dent. Mater..

[8] Quinn J.B., Quinn G.D. (2010). Practical and systematic review of Weibull statistics for reporting the strengths of dental materials. Dent. Mater..

[9] Sacher E., França R. (2019). Dental Biomaterials.

[10] Kim D., Shim J.S., Lee D., Shin S.H., Nam N.E., Park K.H., Shim J.S., Kim J.E. (2020). Effects of post-curing time on the mechanical and color properties of three-dimensional printed crown and bridge materials. Polymers.

[11] de Araujo L.O.F., Barreto O., de Mendonça A.A.M., França R. (2015). Assessment of the degree of conversion in light-curing orthodontic resins with various viscosities. Appl. Adhes. Sci..

[12] Robertson L., Phaneuf M., Haimeur A., Pesun I., França R. (2016). Degree of Conversion and Oxygen-Inhibited Layer Effect of Three Dental Adhesives. Dent. J..

[13] Reymus M., Lümkemann N., Stawarczyk B. (2019). 3D-printed material for temporary restorations: Impact of print layer thickness and post-curing method on degree of conversion. Int. J. Comput. Dent..

[14] O’Neill P.F., Kent N., Brabazon D. (2017). Mitigation and Control of the Overcuring Effect in Mask Projection Microstereolithography. AIP Conference Proceedings.

[15] Choi J.W., Wicker R.B., Cho S.H., Ha C.S., Lee S.H. (2009). Cure-depth control for complex 3D microstructure fabrication using dynamic mask projection microstereolithography. Rapid Prototyp. J..

[16] Willi A., Patcas R., Zervou S.K., Panayi N., Schatzle M., Eliades G., Hiskia A., Eliades T. (2023). Leaching from a 3D-printed aligner resin. Eur. J. Orthod..

[17] Albuquerque P.P., Bertolo M.L., Cavalcante L.M., Pfeifer C., Schneider L.F. (2015). Degree of conversion, depth of cure, and color stability of experimental dental composite formulated with camphorquinone and phenanthrenequinone photoinitiators. J. Esthet. Restor. Dent..

[18] Tahayeri A., Morgan M., Fugolin A.P., Bompolaki D., Athirasala A., Pfeifer C.S., Ferracane J.L., Bertassoni L.E. (2018). 3D printed versus conventionally cured provisional crown and bridge dental materials. Dent. Mater..

[19] Tian Y., Chen C., Xu X., Wang J., Hou X., Li K., Lu X., Shi H., Lee E.S., Jiang H.B. (2021). A Review of 3D Printing in Dentistry: Technologies, Affecting Factors, and Applications. Scanning.

[20] Shin S.H., Lim J.H., Kang Y.J., Kim J.H., Shim J.S., Kim J.E. (2020). Evaluation of the 3D Printing Accuracy of a Dental Model According to Its Internal Structure and Cross-Arch Plate Design: An In Vitro Study. Materials.

[21] Tas H., Demirci F., Tuzlali M., Bahce E., Yildirim Avcu G. (2022). Evaluation of the accuracy of dental casts manufactured with 3D printing technique in the All-on-4 treatment concept. J. Adv. Prosthodont..

[22] Štaffová M., Ondreáš F., Svatík J., Zbončák M., Jančář J., Lepcio P. (2022). 3D printing and post-curing optimization of photopolymerized structures: Basic concepts and effective tools for improved thermomechanical properties. Polym. Test..

[23] Digholkar S., Madhav V.N., Palaskar J. (2016). Evaluation of the flexural strength and microhardness of provisional crown and bridge materials fabricated by different methods. J. Indian Prosthodont. Soc..

[24] Alharbi N., Osman R., Wismeijer D. (2016). Effects of build direction on the mechanical properties of 3D-printed complete coverage interim dental restorations. J. Prosthet. Dent..

[25] Jiang C.-P., Romario Y.S., Bhat C., Hentihu M.F.R., Zeng X.-C., Ramezani M. (2023). Design and fabrication of multi-material pneumatic soft gripper using newly developed high-speed multi-material vat photopolymerization 3D printer. Int. J. Adv. Manuf. Technol..

